# Our New York Letter

**Published:** 1889-08

**Authors:** M. B. Hutchins

**Affiliations:** New York


### OUR NEW YORK LETTER.

New York, July, 1889.

In the summer medical and surgical work in New York is left in the hands of the "assistants," the leading men going away and devoting only a little time to their hospital and private practice. As a consequence the medical and surgical work is less interesting, and there is less which can be reported under the heading "Progress of Medicine and Surgery." All the leading men have one or two "assistants"—generally younger doctors, who are practicing on their own account. These are not prone to lament the arrival of summer and the departure of the "old man," "his nibs," or the "great mogul" (as they may be reverently termed), for the younger men are able to pick up many a crumb which otherwise would not fall in reach—or fall at all. Many of these assistants are remarkably well up, and some fully able to take the place left for them. They may do as good work and as brilliant operations as "the old man," and they get many good cases in summer which they are able to keep. But this summer exodus necessarily causes some stagnation in the hospital and instructive departments. And the Academy, where one goes in hope of hearing the newest and best in our profession, now offers less or no inducement to the seeker after knowledge.

But summer does not close the hospitals ; the work must go on, and that queer genius, the hospital charity case, continues to put in an appearance. Of course there are varieties of patients—some good patients and some, the majority, bad patients. The charity case seems hardest to please, to have no idea of gratitude, and some even get the impression that they are conferring a favor in appearing. In the language of slang, these should receive "the bounce" in lieu of treatment. Hospital physicians soon learn to doubt the tales of distress they hear (just as they ignore all statements made by "specific cases"), and trust to their own investigations altogether. Patients in the hospital delight in smuggling in, or having brought to them, improper articles of food. Within a few hours the doctors hear complaints of peculiar pains, sudden, unaccountable (?) illness, etc. The cause is easily discovered and strict orders given. Often patients can't see how bananas, beer, sausage and such articles can "hurt" them, and are prone to disobey orders at first opportunity. The out-patient classes generally appear fairly well-to-do and do not seem ashamed to accept treatment said to be only for those who are unable to pay a doctor outside. When one is found who confesses ability to pay and receives the doctor's office card, that patient is generally never seen at the doctor's office or at the clinic again. The Irish and German people predominate at the clinics. I have not seen over a half dozen negroes at the skin clinics altogether. A clinic without negroes would be a curiosity in the South, and one might believe that it would be no easy matter to diagnose skin disease in the negro, when he has read the descriptions of the disease only as seen in the white skin, and has seen only a few in the black.

Hospitals are numerous and well patronized in New York, and new ones are constantly heard of. Other and smaller cities have found them necessary, while Atlanta, with her seventy-five thousand inhabitants, remains one of the few which have been content to delay the founding of a hospital which will fill a void now regretfully recognized by those who feel a pride in the city.

There has been, recently, in the Skin and Cancer Hospital a rare case, and one of peculiar interest. Carcinoma attacked the left breast a few years ago. No operation was performed, and the disease "extirpated" the gland completely. Upon entering the patient presented every appearance of one who had been operated upon, and had suffered a recurrence. Over the pectoralis major muscle there was a partially ulcerating, partially cicatricial, contracted sulcus; borders, in places, contracted and nodular. Axillary lymphatics enlarged and indurated. Left arm swollen through obstructed circulation. Scattered over the chest and clavicular regions were nodules in the skin, varying from the size of a pea to that of a hazelnut. The right mammary gland presented carcinomatous induration on its outer side. Patient is comparatively strong, always in good spirits and seems capable of fighting death for some time yet. Considered herself well enough to leave the hospital and has gone home. Operation impracticable.

In broken down, offensive, cancerous growths creolin, used in solution of one per cent., demonstrates its utility as a disinfectant. Surfaces remain clear, odor is destroyed, and the ulceration even seems to be checked. A dressing, kept constantly wet with this solution, was the local treatment for the above case. A man who entered the hospital with an acute general eczema was dressed with an ointment of salicylic acid and oxide of zinc, the salicylic being but gr. x to ℥i. In about a week the patient developed rather peculiar and somewhat alarming symptoms. Pulse tended to remain near 120, or to go even higher; temperature was normal. Attacks would occur, in which patient complained of heat and fullness in head; throbbing of carotids noticed, and nausea was present. The urine was tested when patient first entered, but nothing abnormal was observed. The legs were somewhat œdematous, but this is not rare in acute eczema of the feet and legs. There was a history of "fainting spells," and of patient having been overcome by heat once. Symptoms puzzled those who saw the man, until the test apparatus and the microscope were again employed. Urine was found to contain muco-pus, specific gravity 1015, and albumen quite abundant. Put in a settling glass, and the sediment then examined microscopically, granular casts, hyaline casts, and a few red blood cells were seen. Upon some of the casts were epithelial cells, such as are described as coming from the convoluted tubules. Crystals of urate of sodium alone were seen, though ammonical decomposition seemed to have had time to take place. There are two points which make the case worth the description. The value of the test apparatus and microscope in assisting the physician in diagnosis is one, and the other is the question as to the thick application of ointment and bandaging having obstructed perspiration sufficiently to produce the symptoms described. Or was it possible that the treatment or condition of the patient in some way excited a latent diseased condition of the kidneys? The patient now has many symptoms of Bright's disease and uraemic poisoning. Milk diet was tried upon a man with albuminuria without the slightest diminution in quantity of albumen.

A little girl, aged sixteen, with congenital syphilis, was given "mixed treatment" containing only gr. v. of potassium iodide to the dose. This was taken three times per day, and within four days there appeared upon the face a number of dome-shaped lesions, some being larger than the thumb nail. The lesions were sharply defined, semi-indurated and covered with pin-head sized, pustular points. Later they became crusted, crusts being partly composed of blood. Treatment was stopped, and the eruption began to decline at once, no new lesions appearing. In the majority of the new formations the only trace left was a flat, red, lightly crusted spot. A few on chin left pyramidal, very hard, pea-sized tubercles, which still persist over a month after the outbreak. The iodide unquestionably caused the eruption, though none of the descriptions which I have seen in the books include lesions with the above characteristics. Yet the iodides are described as capable of producing every kind of the lesions likely to be seen on the skin when diseased.

The case of purpura rheumatica described in a former letter is just now (apparently) well. Has had a number of relapses, occurring on the legs, the result of leaving her bed and exercise. These later hemorrhagic spots occurred most numerously at point of compression by the garter, forming a perfectly straight line partially encircling the leg. The position of the garter was changed; a new line appeared at the new location of the garter. Blood, unmixed with the stools, was discharged from the rectum, showing purpuric condition of intestinal mucous membrane. This was treated with a prescription containing tannic acid, and did not recur. Fluid extract of ergot exerted no influence upon the disease, but tinct. of the chloride of iron seemed beneficial.

Patient has constant rheumatic pains and the heart gives evidence of organic disease.

Usually the urine of every hospital patient is tested when they enter the hospital. Microscopic examinations only occasional. During the past four months albumen was found in the urine of only three cases and sugar not at all. One case had acute eczema with tendency to furuncular outbreaks; the second had acute eczema; and the third was supposed to have carcinoma of the liver. For the past three or four months glycerine, by the mouth, by enema and by suppository, has been tried here in a number of cases of constipation. While pleasant to take and seemingly free of tendency to the so common "griping" of laxatives and cathartics, the conclusion is that glycerine, in almost any dose, small or large, given alone, is unreliable.

Ungt. Hg. Ammoniat. applied in full strength to ulcerating syphilitic areas, which were very irritable from previous treatment, produced no pain, and the process of healing was quite rapid—though emplastrum hydrarg. acted better upon similar surfaces on the same patient.

M. B. HUTCHINS, M. D.

